# Special Issue “Molecular Mechanisms of Allergy and Asthma: 3rd Edition”

**DOI:** 10.3390/ijms262411928

**Published:** 2025-12-11

**Authors:** Bilal Alashkar Alhamwe, Daniel P. Potaczek

**Affiliations:** 1 College of Pharmacy, International University for Science and Technology (IUST), Daraa 15, Syria; 2 Translational Inflammation Research Division & Core Facility for Single Cell Multiomics, Medical Faculty, Philipps-University of Marburg, 35043 Marburg, Germany; 3 Center for Infections and Genomics of the Lung (CIGL), Justus Liebig University, 35390 Giessen, Germany

Over the past few decades, there has been a steady increase in the prevalence of allergic diseases worldwide. Asthma, allergic rhinitis, atopic dermatitis, and food allergies are among the most common chronic diseases in children and adolescents. In addition, they often persist into adulthood. Therefore, allergic diseases are not only associated with a significant reduction in the health and quality of life of patients, but also represent a serious global socio-economic burden. In this context, the need to develop better strategies for the prevention, diagnosis, and treatment of these diseases is obvious [1,2,3]. To this end, it is necessary to gain an even deeper understanding of the mechanisms underlying allergic disorders, which, in many cases, overlap with mechanisms more commonly found in other branches of biomedicine. The original and review articles included in this Special Issue report on the latest discoveries in these areas.

The perinatal period plays a pivotal role in the development of allergic disorders later in life [4,5]. Accordingly, the article by Grijincu et al. (Contribution 1) provides an overview of the mechanisms involved in allergic sensitization and the progression of allergic diseases, with the aim of identifying which prenatal risk and protective factors influence the development of allergic conditions and how. It has been shown that a significant number of babies present transiently with low protein kinase C zeta (PKCζ) levels in cord blood T cells (CBTC), associated with reduced ability to transition from a neonatal T helper 2 cell (Th2) to a mature Th1 cytokine bias, leading to a higher risk of developing allergic sensitization, compared to neonates whose T cells have ‘normal’ PKCζ levels [6,7]. Now, Perveen and Ferrante (Contribution 2) expand those observations by showing that cord blood monocytes and neutrophils can also display immaturity or deficiency of several PKC isozymes, extending the concept of “perinatal PKC dysregulation” beyond T cells.

Toll-like receptors (TLRs) of the human immune system are specialized pathogen detectors able to link innate and adaptive immune responses. TLRs, whose ligands include microorganism-derived compounds such as lipids, lipo- and glycoproteins, have been involved in the pathogenesis of allergic disorders [8,9]. The study by Devulder et al. (Contribution 3) demonstrates that pulmonary administration of FSL-1 (Pam2CGDPKHPKSF), a TLR2/6 agonist, reduced airway hyperresponsiveness and eosinophilic inflammation, and also induced the recruitment of natural killer cells in lung parenchyma in an ovalbumin (OVA)-based murine model of allergic airway inflammation mimicking human asthma. Rainer and coworkers (Contribution 4) characterized, in turn, the immune-modulating properties of different β-glucans, which are naturally derived sugar structures and components of dietary fibers that activate C-type lectin receptors (CLRs), TLRs, and complement receptors (CRs), on myeloid dendritic cells. Based on these results, β-glucans might represent interesting adjuvants for future allergy treatment, with distinct functional profiles that may help fine-tune immune responses.

In their systematic review, Browne et al. (Contribution 5) evaluated the current evidence for the involvement of epithelial-derived extracellular vesicles (EVs) in immunoglobulin E (IgE)-mediated allergic sensitization. Indeed, there is growing evidence for the contribution of EVs and their cargo, especially microRNAs (miRNAs) in the pathogenesis of allergic disorders and asthma [10,11]. Importantly, Browne and colleagues highlight barrier-specific effects and the lack of studies on skin-derived EVs, representing a notable knowledge gap. AllergoOncology is a quickly developing field oriented towards cross-disciplinary insights in relationships between allergic diseases and cancers [12,13,14]. Alashkar Alhamwe et al. (Contribution 6) expanded our knowledge in this highly interesting research area by finding that peripheral inflammation featuring eosinophilia or neutrophilia is associated with the survival and infiltration of eosinophils within the tumor in certain histological subgroups of patients with non-small cell lung cancer. These observations refine current views on how type 2 (T2)–skewed inflammation interacts with the tumor microenvironment. Interestingly enough, there are studies showing that cells typically associated with allergy, specifically mast cells, contribute to tumor growth under stimulation with EVs deriving from pancreatic cells [15].

De Vivero and coworkers (Contribution 7) investigated the complexity of T2 responses, as measured by the exhaled fraction of nitric oxide (FeNO) and other biomarkers of T2 inflammation [16,17], among asthmatics and subjects with helminth parasitic infections. Among others, they found that FeNO levels are not influenced by either natural exposure to helminth parasites or active infection, which supports its usefulness as a robust asthma biomarker in the tropics. Their study also highlights lung-specific regulatory mechanisms in helminth-exposed but otherwise healthy children. Therefore, the study adds to our understanding of the complex interaction between helminths and allergy [18,19].

In allergology, molecular characterization of allergens and immunological identification of high (or low) allergenic substances is crucial [20]. However, determination of the in vivo sensitizing potential constitutes next step of those investigations. In this Special Issue, Donado et al. (Contribution 8) assessed sensitizing abilities of *Dermatophagoides pteronyssinus* allergen Der p 23. They found Der p 23 to be a frequent IgE sensitizer in humans, as well as an effective inducer of allergic airway inflammation in mice. This combined clinical–experimental approach underscores the translational significance of Der p 23.

Finally, Oprițescu and colleagues (Contribution 9), investigated clinical and immunological correlations of serum IgE and IgA levels in the context of infectious disease in pediatric patients with Henoch–Schönlein Purpura. Curiously, children with infectious diseases consistently exhibited elevated IgE levels and normal IgA levels during treatment despite no identified sensitization to any allergens, alongside an increased risk of disease recurrence. In light of these highly interesting observations, further research on this topic is required. These findings again suggest that IgE-associated immune patterns may contribute to disease dynamics even in conditions not classically considered allergic.

In summary, this Special Issue contains a series of articles presenting intriguing research findings on allergies and asthma and their underlying mechanisms. Together, these contributions illustrate how allergic inflammation intersects with perinatal immunology, microbial sensing, epithelial biology, oncology, parasitology, and molecular allergology. This Special Issue will be continued in its fourth edition in the International Journal of Molecular Sciences and Allergies journals.
